# MiR-383-5p inhibits the proliferation and migration of lung adenocarcinoma cells by targeting SHMT2

**DOI:** 10.7150/jca.89733

**Published:** 2024-03-17

**Authors:** Xianxia Bi, Luwei Wang, Hua Li, Ying Ma, Ruoyu Guo, Jicheng Yue, Lijun Kong, Xiangqian Gong, Fei Jiao, Eugene Chinn, Jinxia Hu

**Affiliations:** 1 Peninsula Cancer Research Center of Binzhou Medical University, YanTai, Shandong 264003, P.R. China.; 2 Yantai Environmental Sanitation Management Center, YanTai, Shandong 264000, P.R. China.; 3 Department of Biochemistry and Molecular Biology, Binzhou Medical University, YanTai, Shandong 264003, P.R. China.; 4 Department of Gastrointestinal Surgery, Yuhuangding Hospital, YanTai, Shandong 265499, P.R. China.

**Keywords:** miR-383-5p, SHMT2, lung adenocarcinoma, proliferation, migration

## Abstract

**Purpose:** To explore the effects of miR-383-5p and serine hydroxymethyltransferase 2 (SHMT2) on the proliferation and migration of lung adenocarcinoma cells.

**Methods:** SHMT2 expression in lung adenocarcinoma and normal tissues was investigated using The Cancer Genome Atlas database. Immunohistochemical analysis was performed to confirm SHMT2 expression in lung adenocarcinoma and adjacent normal lung tissues. Bioinformatics analysis and luciferase reporter assays were used to analyze the relationship between miR-383-5p and SHMT2 expression. The protein expression levels of SHMT2, vimentin, N-cadherin, E-cadherin, Bcl-2, and cyclinD1 were analyzed using western blotting. The reverse transcription-quantitative polymerase chain reaction was used to detect SHMT2 knockdown efficiency, miR-383-5p overexpression, and inhibition efficiency. The proliferative ability of cells was detected using the Cell Counting Kit-8 assay. The Transwell assay was used to detect the migration ability of cells.

**Results:** SHMT2 expression was significantly increased in patients with lung adenocarcinoma compared to that in control patients; the higher the SHMT2 expression the worse the outcomes were in patients with lung adenocarcinoma. SHMT2 knockdown inhibited the proliferation, migration, and epithelial-mesenchymal transition of lung adenocarcinoma A549 and H1299 cells. MiR-383-5p directly targeted and downregulated SHMT2 in A549 and H1299 cells. The effects of miRNA-383-5p on the proliferation and migration of these cells differed from those of SHMT2. Exogenous overexpression of SHMT2 reversed the miR-383-5p-induced proliferation and migration inhibition in A549 and H1299 cells.

**Conclusion:** MiR-383-5p inhibits the proliferation and migration of lung adenocarcinoma cells by targeting and downregulating SHMT2.

## Introduction

According to the Global Cancer Report released by the International Agency for Research on Cancer of the World Health Organization, there were approximately 2.2 million new cases and 1.8 million deaths from lung cancer worldwide in 2020, ranking first in the number of cancer-related deaths. Lung adenocarcinoma (LUAD) is the most common subtype of lung cancer, accounting for approximately 40% of primary lung tumors 1. It often occurs in women and smokers and has the highest mortality rate among all lung cancers. Moreover, LUAD has no clinical manifestations in its early stages and is difficult to detect. Metastasis usually occurs in the middle and late stages, although many patients with LUAD develop metastasis in the early stages. Despite substantial achievements in studies on the pathogenesis of LUAD 2, 3, its global incidence and mortality rate are still increasing. Therefore, identifying targets for treating LUAD is important.

MicroRNAs (miRNAs) are single-stranded non-coding RNAs of approximately 20-24 nucleotides in length. MiRNAs target the 3ʹ-untranslated region (UTR) of mRNA and play crucial roles in various biological processes, including cell metabolism 4, 5, proliferation 6, and apoptosis 7, 8. Studies have shown that miR-383-5p acts as a tumor suppressor that regulates the expression of downstream target genes 9-11.

The serine and glycine synthetic pathways are involved in the biosynthesis of cellular components that maintain cell proliferation 12. Serine hydroxymethyltransferase 2 (SHMT2) is a key enzyme in one-carbon unit metabolism 13. This enzyme catalyzes the conversion of serine and tetrahydrofolate to glycine and 5,10-methylenetetrahydrofolate, which ultimately supports the synthesis of thymidine and purines, and promotes tumor growth 14. Elevated SHMT2 expression has been associated with poor prognosis in many cancerous tumors 15, 16. SHMT2 plays a crucial role in human carcinogenesis, highlighting a potential regulatory mechanism that promotes tumor progression. Therefore, it can be used as a prognostic marker and target for anticancer therapy.

In this study, we investigated the role of SHMT2 in the proliferation and migration of LUAD cells and the relationship between miR-383-5p and SHMT2. Our findings may be of great value in the development of targeted treatments for lung cancer in the future.

## Materials and methods

### Cell lines, cell culture, and reagents

293T cells, Normal lung BEAS-2B epithelial cells and the human lung cancer cell lines A549 and H1299 were purchased from the American Type Culture Collection (Manassas, VA, USA). Cells were cultured in Roswell Park Memorial Institute 1640 (Pricella, Wuhan, China) complete medium containing 10% fetal bovine serum (Pricella) and 1% penicillin-streptomycin solution (Pricella) in a humidified cell incubator at 37 °C and 5% CO_2_.

### Human tissue immunohistochemistry

Samples of carcinomas and adjacent normal tissues were obtained from surgical specimens of patients with lung cancer at Yantaishan Hospital (Yantai, Shandong Province, China) and embedded in paraffin. All studies were approved by the Ethics Committee of Binzhou Medical University, and informed consent was obtained from all patients.

Immunohistochemistry was performed according to routine operations. The sections were subjected to deparaffinization and antigen retrieval with 10 mM sodium citrate (pH 6.0) solution and blocked with phosphate-buffered saline (PBS) containing 5% goat serum (Zhong Shan Golden Bridge Biotechnology Company, Beijing, China) at room temperature (∼25 °C) for 30 min. The primary antibody was diluted in PBS containing 5% goat serum (1:200), added to the slides, and incubated overnight at 4 °C, followed by incubation with the horseradish peroxidase-conjugated secondary antibody and staining with the 3.30-diaminobenzidine substrate (Zhong Shan Golden Bridge Biotechnology Company).

### MiRNAs and siRNA

Both small interfering (si)RNA and miRNAs were purchased from GenePharma (Shanghai, China).

The siRNA and miRNA sequences were:

*miR-383-5p* mimic sense: 5′-AGAUCAGAAGGUGAUUGUGGCU-3′

antisense: 5′-CCACAAUCACCUUCUGAUCUUU-3′

*miR-383-5p* inhibitor sense: 5′-AGCCACAAUCACCUUCUGAUCU-3′

*SHMT2* siRNA sense: 5′-GUGAUUCCCUCGCCUUUCATT-3′

antisense: 5′-UGAAAGGCGAGGGAAUCACTT-3′

### Plasmids and cell transfection

The pmirGLO-SHMT2 3ʹ-UTR WT plasmid was cloned using a homologous recombination kit (Vazyme, Nanjing, China). The cloning primers used were as follows:

pmirGLO-*SHMT2* 3ʹ-UTR-forward:

5′-GTGTAATTCTAGTTGTTTAAAAGGCACCTGGGAAATGAGGC-3′

pmirGLO-*SHMT2* 3ʹ-UTR-reverse:

5′-CAGGTCGACTCTAGACTCGAGTGGGCAAAATACAATTTCATTTAAC-3′

The recombinant PCR primers were:

pmirGLO-*SHMT2* 3′-UTR Mut-forward:

5′-TGCAGCCTCCTCTTTCTGTCGAGCCGACACCAGACGTGAT-3′

pmirGLO-*SHMT2* 3′-UTR Mut-reverse:

5′-ATCACGTCTGGTGTCGGCTCGACAGAAAGAGGAGGCTGCA-3′

The HA-SHMT2 plasmid was cloned using a homologous recombination kit (Vazyme); the linearized carrier was linked to the target fragment, and the cloning primers used were as follows:

HA-forward: 5′-CGGCCGCGGGGATCCAGA-3′

HA-reverse: 5′-TTCGGGCCTCCATGGCCA-3′

*SHMT2*-forward:

5′-TATGGCCATGGAGGCCCGAAATGCTGTACTTCTCTTTGTTTTGGG-3′

*SHMT2*-reverse:

5′-TGTCTGGATCCCCGCGGCCGTCAATGCTCATCAAAACCAGGC-3′

Lipofectamine 2000 (Thermo Fisher Scientific, Shanghai, China) was used for transfection.

### Western blotting

Cells were lysed with radioimmunoprecipitation assay lysis buffer (Solarbio, Beijing, China) containing 1% phenylmethylsulfonyl fluoride (Solarbio), separated by sodium dodecyl sulfate-polyacrylamide gel electrophoresis, transferred to polyvinylidene difluoride membranes, and incubated with primary and secondary antibodies. SHMT2, N-cadherin, E-cadherin, and β-actin antibodies were purchased from Cell Signaling Technology (Danvers, MA, USA). Vimentin (5741S) was purchased from Cell Signaling Technology and vimentin (60330-1-lg) was purchased from Proteintech (Wuhan, China). The Bcl-2 antibody was purchased from Bioworld (Nanjing, China), and the cyclinD1 antibody was purchased from Bioss (Beijing, China).

### Reverse transcription-quantitative polymerase chain reaction (RT-qPCR)

Cells were collected, and total RNA and miRNA were extracted using a cell miRNA extraction kit (Vazyme). Reverse transcription reagents (Vazyme) were then used to convert the total RNA into cDNA. The miRNA 1st Strand cDNA Synthesis Kit (Vazyme) was then used to convert the miRNA into cDNA. The miRNA reverse transcription primers were designed using the miRNA Design V1.01 software (Vazyme) (primer sequence is shown below). The relative expression levels of SHMT2 and miR-383-5p were assessed using the 2^-ΔΔCt^ method.

The supplementary primer sequences used were as follows:

*SHMT2*-forward: 5′-CCTGCCCTGAGTTTCCATTA-3′

*SHMT2*-reverse: 5′-GTCTGGTGTCGGCTCTGAT-3′

*β-actin*-forward: 5′-CAGCCTTCCTTCTTGGGTAT-3′

*β-actin*-reverse: 5′-TGGCATAGAGGTCTTTACGG-3′

*miR-383-5p* microRNA sequence: 5′-AGAUCAGAAGGUGAUUGUGGCU-3′

Reverse transcription primer sequences: 5′-GTCGTATCCAGTGCAGGGTCCGAGGTATTCGCACTGGATACGACAGCCAC-3′

*miR-383-5p*-forward: 5′-GCGCGAGATCAGAAGGTGATT-3′

*miR-383-5p*-reverse: 5′-AGTGCAGGGTCCGAGGTATT-3′

*U6-forward:* 5′-CTCGCTTCGGCAGCACA-3′

*U6-reverse:* 5′-AACGCTTCACGAATTTGCGT-3′

### Luciferase reporter gene detection

The predicted putative binding site of miR-383-5p on the wild-type (WT) SHMT2 3′-UTR was cloned into the pmirGLO dual luciferase miRNA target expression vector (pmirGLO-SHMT2 3′-UTR WT). The mutant (Mut) pmirGLO-SHMT2 3′-UTR (pmirGLO-SHMT2 3′-UTR Mut) was produced by recombinant PCR.

293T cells were inoculated into 24-well plates and transfected with a WT or Mut *SHMT2* 3′-UTR reporter and the miR-383-5p mimic or negative control using Lipofectamine 2000 when the cell density reached 70% confluence. Transfected cells were harvested, and a luciferase reporter gene assay was performed according to the instructions of the firefly luciferase reporter assay kit (Beyotime, Shanghai, China).

### Cell proliferation assay

The cells were seeded into 96-well plates at a density of 2 × 10^3^ cells/well. The Cell Counting Kit-8 (CCK-8) solution (10 µL; Absin, Shanghai, China) was added at 0 (approximately 4 h after inoculation and cell adhesion), 24, 48, 72, and 96 h. The cells were cultured for 2 h after adding the CCK-8 solution and the absorbance at 450 nm was measured using a microplate spectrophotometer.

### Migration assay

The upper chamber of the Transwell plate was inoculated with 2 × 10^4^ cells/well in serum-free medium. Then, medium containing 30% serum was added to the lower chamber. The upper chamber was slowly placed into the lower chamber and incubated for 24 h. The chambers were then washed twice with PBS, fixed with 4% paraformaldehyde for 30 min, washed again with PBS, gently wiped with a cotton swab, stained with crystal violet for 20 min, washed three more times with PBS, and dried at room temperature. Randomly selected fields were photographed under an inverted microscope (Leica, Wetlzar, Germany).

### Apoptosis detection

Forty-eight hours after transfection, the cells were treated according to the instructions of the Annexin Ⅴ-FITC/PI apoptosis detection kit (Beyotime) and then detected by flow cytometry and analyzed using the FlowJo software (version 10.6.2; BD Biosciences, Franklin Lakes, NJ, USA).

### Statistical analyses

Experimental data are presented as the mean ± standard deviation. T-tests were used to compare the mean values between two groups. A one-way analysis of variance was used for comparisons between multiple groups, followed by a post-hoc Tukey test for multiple comparisons. Prism 9 software (GraphPad, La Jolla, CA, USA) was used for statistical analyses. *P* < 0.05 was considered statistically significant. The in vitro experiments were performed in triplicate and repeated three times.

## Results

### SHMT2 is upregulated in LUAD and is a prognostic factor for a poor outcome

**Figure 1:**
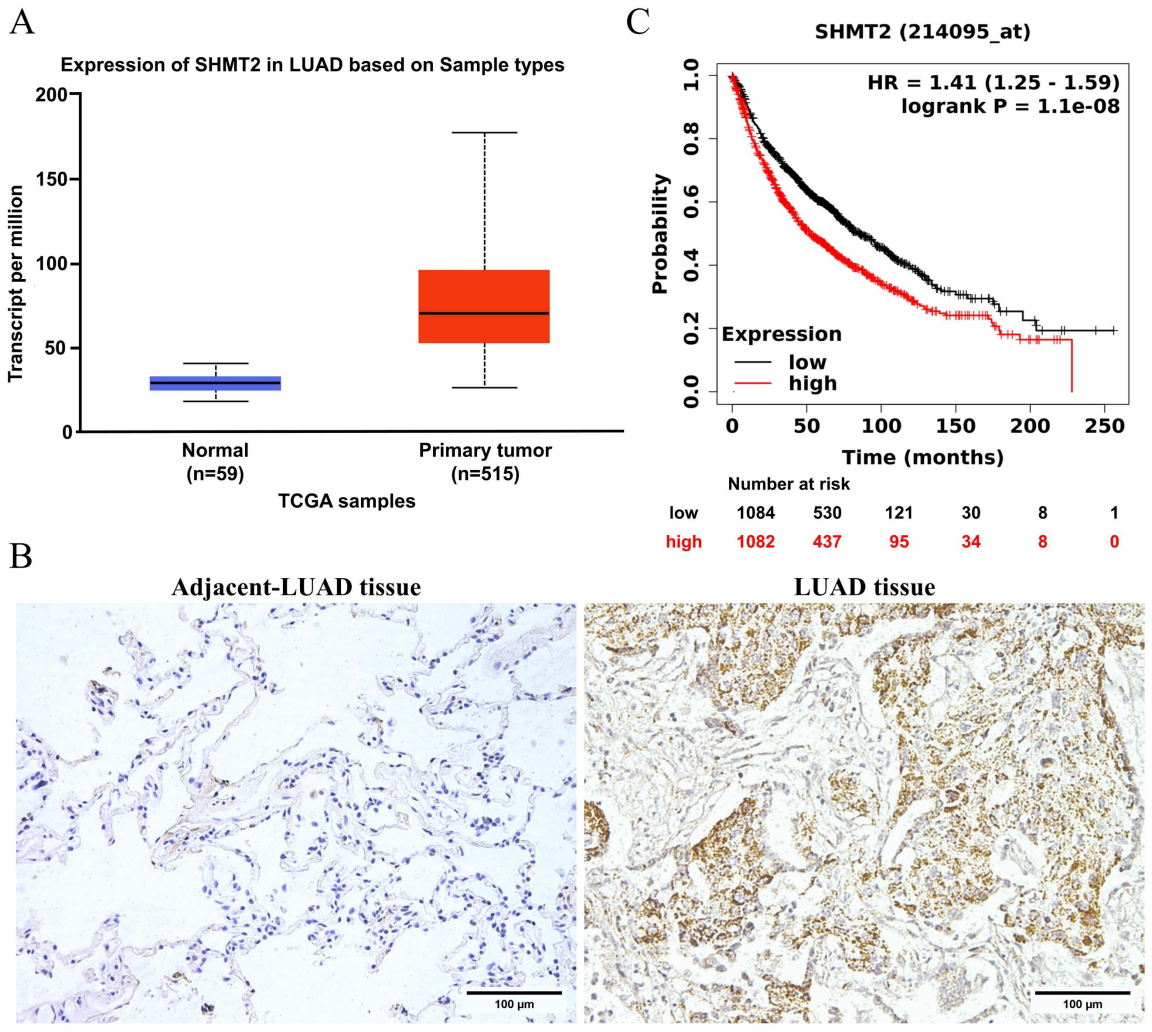
** Serine hydroxymethyltransferase 2 (SHMT2) is upregulated and is a prognostic factor in patients with lung adenocarcinoma (LUAD). A.** SHMT2 expression in LUAD tissues from The Cancer Genome Atlas was analyzed using the UALCAN website. **B.** Immunohistochemical staining of SHMT2 in LUAD and adjacent tissues. **C.** Kaplan-Meier overall survival curve of LUAD patients with different levels of SHMT2 mRNA expression.

To determine the role of SHMT2 in lung cancer, we first investigated its clinical significance in LUAD. According to The Cancer Genome Atlas (TCGA) database, SHMT2 expression in LUAD tissues was significantly increased compared with that in normal tissues (Figure 1A). To verify this result, we performed immunohistochemical staining for SHMT2 in LUAD and adjacent normal lung tissues. This staining showed that SHMT2 expression was significantly increased in LUAD tissues compared with adjacent normal lung tissues (Figure 1B). The extent of SHMT2 expression was correlated with a poor outcome of patients with LUAD, showing that SHMT2 expression was a prognostic factor (Figure 1C). Collectively, these results suggest that SHMT2 expression is clinically significant in LUAD.

### MiR-383-5p directly targets SHMT2

**Figure 2:**
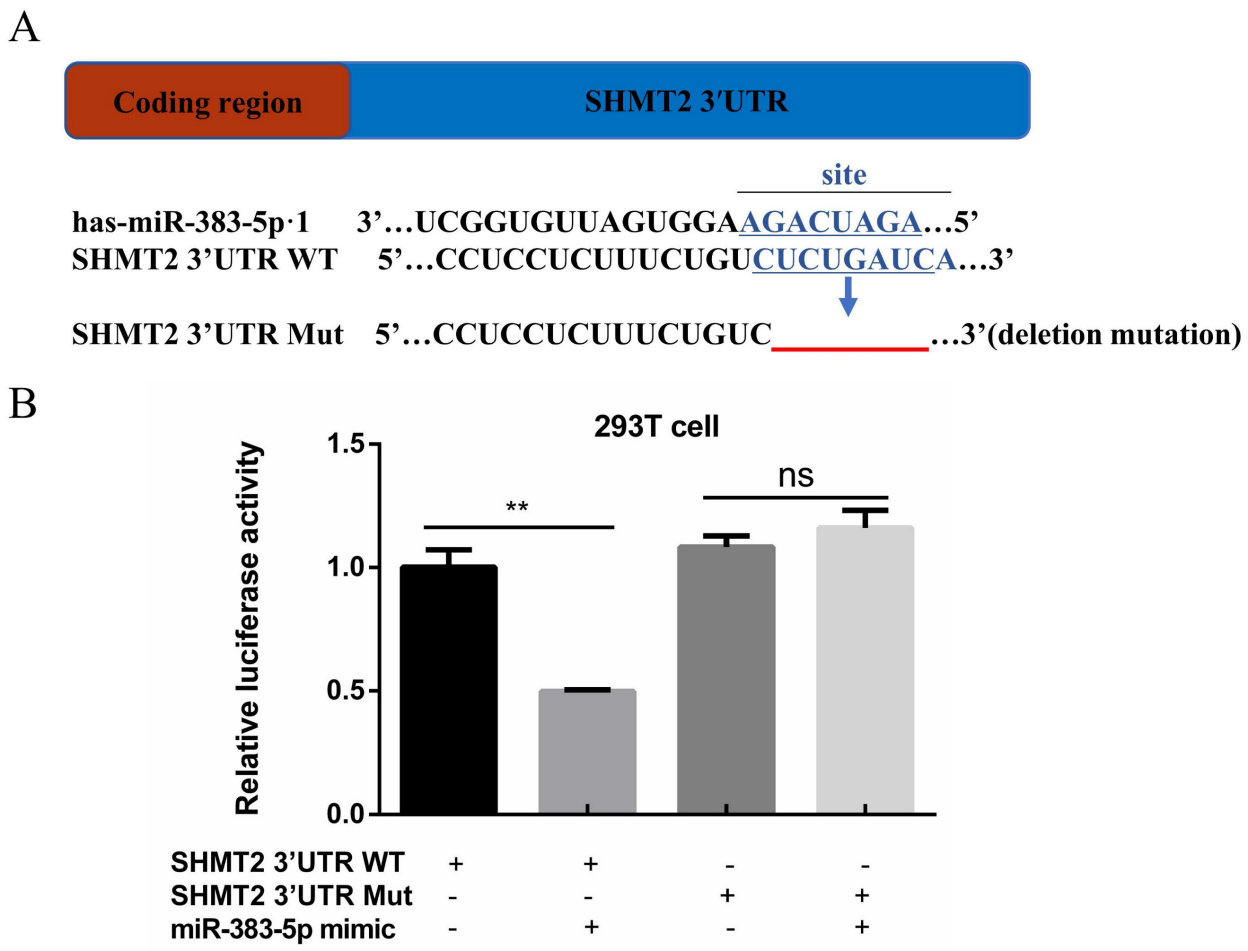
** Relationship between serine hydroxymethyltransferase 2 (SHMT2) and miR-383-5p. A.** The predicted binding site of miR-383-5p in the human SHMT2 3′-untranslated region (UTR). Wild-type (WT) and mutant (MuT) SHMT2 3ʹ-UTR-fused dual luciferase reporters were constructed. Deletion mutations made to the WT SHMT2 3′-UTR are shown by the red line. **B.** Relative luciferase activity assay of the WT and MuT SHMT2 3ʹ-UTR-fused dual luciferase reporters in 293T cells expressing control or an miR‑383‑5p mimic. **P < 0.01.

A TargetScan database query revealed that miR-383-5p could bind to the SHMT2 3′-UTR (Figure 2A). This suggested that SHMT2 may be the downstream target of miR-383-5p. Based on this finding, we inserted the SHMT2 3′-UTR containing the putative binding site of miR-383-5p into the pmirGLO luciferase reporter system vector. We then mutated it with recombinant PCR and performed the luciferase reporter gene assay. The results showed that, compared with cells co-transfected with SHMT2 3′-UTR Mut and the miR-383-5p mimic, cells co-transfected with SHMT2 3′-UTR WT and the miR-383-5p mimic had significantly decreased luciferase activity (Figure 2B). This suggests that miR-383-5p targets SHMT2.

### Knockdown of SHMT2 inhibits the proliferation and migration of LUAD cells

**Figure 3:**
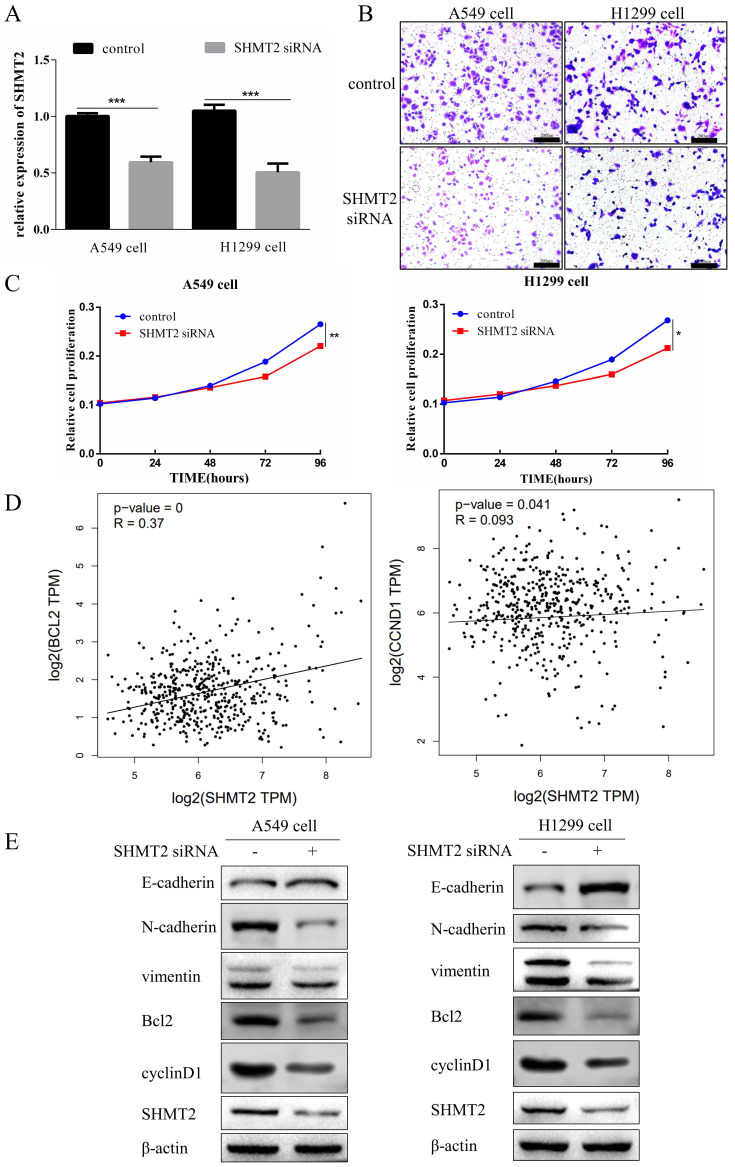
** Serine hydroxymethyltransferase 2 (SHMT2) knockdown inhibits the proliferation and migration of lung adenocarcinoma cells. A.** RT-qPCR results showing the efficiency of SHMT2 knockdown in A549 and H1299 cells. **B.** Migration of A549 and H1299 cells was detected using the Transwell assay. **C.** Proliferation of A549 and H1299 cells was determined using the Cell Counting Kit-8 assay. **D.** Correlation analyses of SHMT2 with Bcl-2 and cyclinD1 using the Gene Expression Profiling Interactive Analysis website. **E.** Western blot analyses of SHMT2 and the indicated proteins in A549 cells and H1299 cells transfected with siRNA targeting SHMT2. *P < 0.05, **P < 0.01, *** *P* < 0.001.

*SHMT2* is an oncogene that promotes cancer cell proliferation and migration 17. Therefore, we investigated the effect of SHMT2 on the proliferation and migration of LUAD A549 and H1299 cells that were transfected with the blank control or SHMT2 siRNA. RT-qPCR revealed that the knockdown efficiency of SHMT2 siRNA was statistically significant (Figure 3A). The results of the CCK-8 cell proliferation analysis and Transwell migration experiments showed that SHMT2 knockdown significantly inhibited the proliferation and migration of A549 and H1299 cells (Figure 3B, C).

To explore the mechanism by which SHMT2 regulates cell proliferation, we analyzed the relationship of SHMT2 with apoptosis- and cell cycle-related genes in the Gene Expression Profiling Interactive Analysis (GEPIA) website. The results showed that SHMT2 was positively associated with Bcl-2 and cyclinD1 (Figure 3D). Then, we examined the effects of SHMT2 on expression of the Bcl-2 and cyclinD1 proteins in A549 and H1299 cells. The results showed that knocking down SHMT2 reduced the expression of both proteins (Figure 3E). The epithelial-mesenchymal transition (EMT) is associated with cell migration; therefore, we examined the effect of SHMT2 on the expression of EMT-related proteins in A549 and H1299 cells. The results showed that *SHMT2* knockdown increased expression of the epithelial marker, E-cadherin. However, the mesenchymal markers, N-cadherin and vimentin, were downregulated (Figure 3E). In summary, *SHMT2* knockdown inhibited the EMT.

### Effect of miR-383-5p on SHMT2 expression in LUAD cells

**Figure 4:**
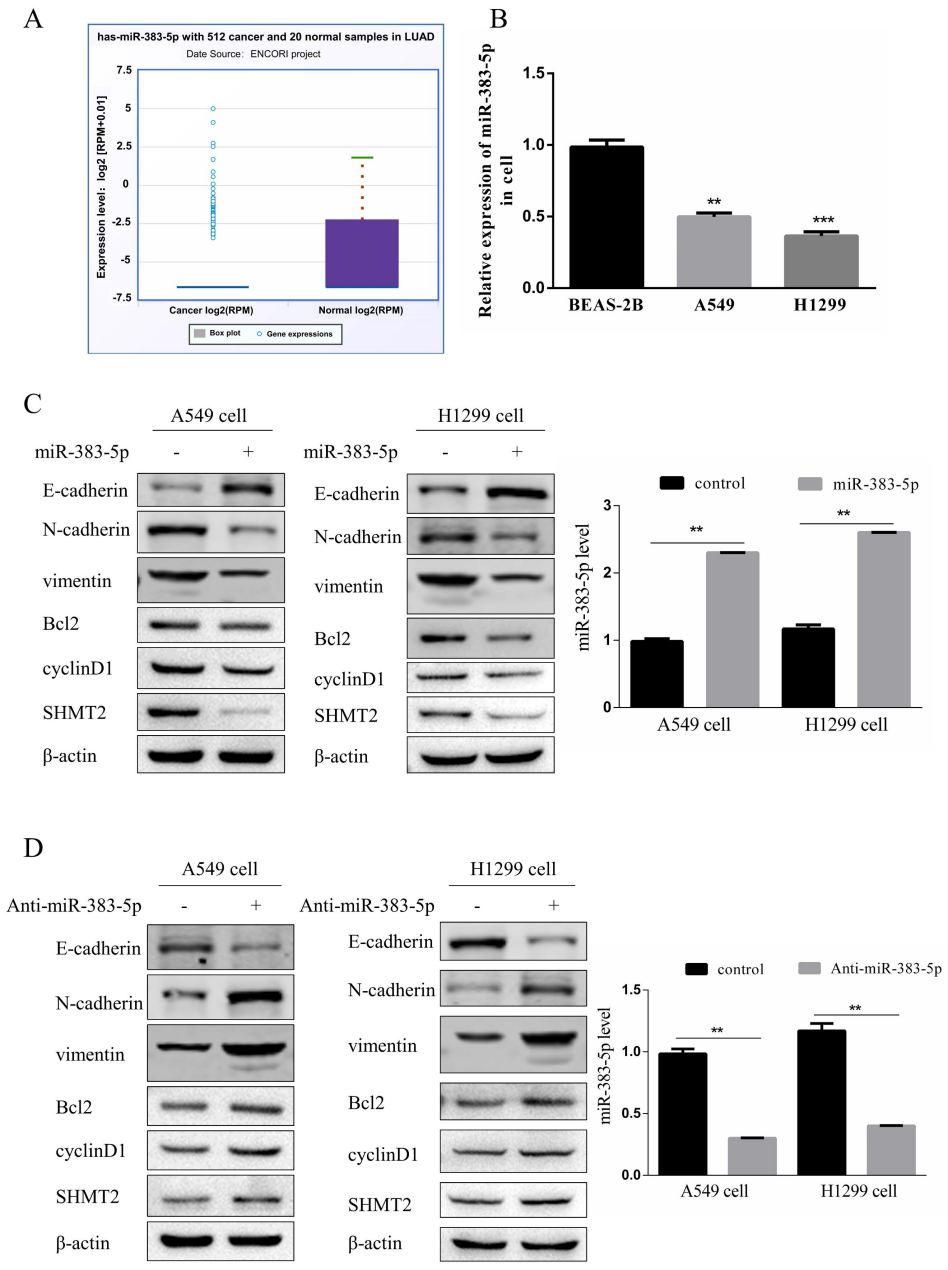

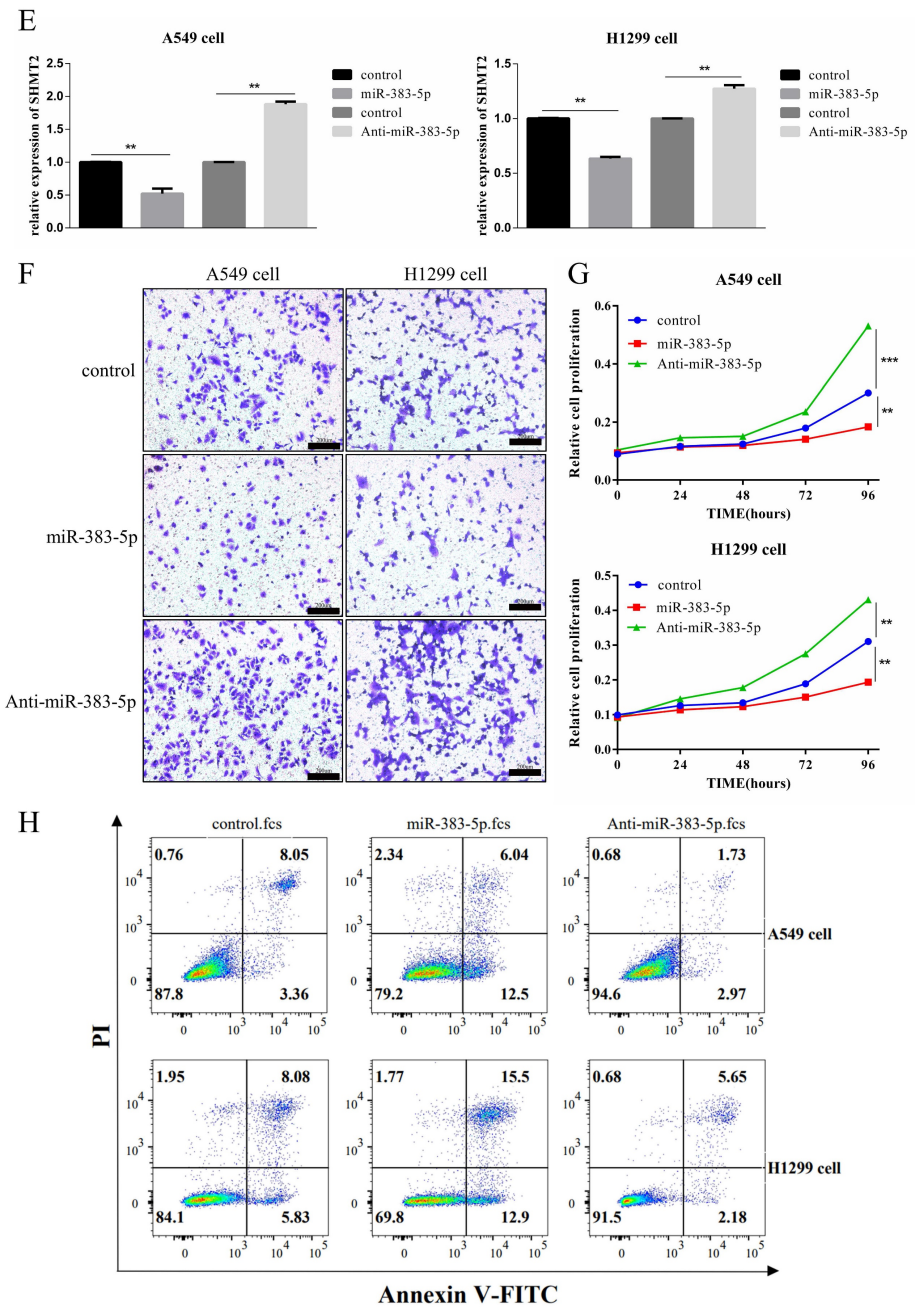
** MiR-383-5p regulates serine hydroxymethyltransferase 2 (SHMT2) expression and affects the proliferation and migration of A549 and H1299 cells. A.** The Encyclopedia of RNA Interactomes (ENCORI/starBase) database was used to analyze the relative expression of miR-383-5p in lung adenocarcinoma and normal tissues. **B.** The relative expression of miR-383-5p in BEAS-2B, A549, and H1299 cells was determined using RT-qPCR. **C.** A549 and H1299 cells were transfected with a blank control or an miR-383-5p mimic. The transfection efficiency of miR-383-5p overexpression was determined using RT-qPCR. Western blotting was used to detect the expression of SHMT2, apoptosis-related proteins, cyclinD1, and epithelial-mesenchymal transition-related proteins using β-actin as the control. **D.** A549 and H1299 cells were transfected with a blank control or an miR-383-5p inhibitor (anti-miR-383-5p). Western blotting was used to detect the expression of SHMT2 and the indicated proteins using β-actin as the control. miR-383-5p levels were detected using RT-qPCR. **E.** The mRNA expression of *SHMT2* was detected using RT-qPCR. **F.** Migration of A549 and H1299 cells was detected using the Transwell assay. **G.** Proliferation of these two lung cancer cell lines was detected using the Cell Counting Kit-8 assay. **H.** Apoptotic cells were detected using flow cytometry and analyzed with FlowJo software. **P < 0.01, *** P < 0.001.

MiR-383-5p reportedly acts as a tumor suppressor 11. According to the Encyclopedia of RNA Interactomes (ENCORI/starBase) database, miR-383-5p expression in LUAD tissues is significantly reduced compared to that in normal tissues (Figure 4A). We detected the expression of miR-383-5p in normal lung BEAS-2B epithelial cells, and lung A549 and H1299 adenocarcinoma cells. The results showed that the expression of miR-383-5p in lung adenocarcinoma cells was significantly lower than that in BEAS-2B cells (Figure 4B). However, the biological function of miR-383-5p in LUAD remained unclear.

*SHMT2* is a target gene of miR-383-5p and its knockdown inhibited the proliferation and migration of LUAD cells. Therefore, we investigated the effect of miR-383-5p on SHMT2 expression in A549 and H1299 cells transfected with a control or miR-383-5p mimic. Western blotting and RT-qPCR showed that the overexpression of miR-383-5p downregulated SHMT2 (Figure 4C, E), and reduced the proliferation and migration abilities of these cells (Figure 4F, G). In contrast, transfection of A549 and H1299 cells with an miR-383-5p inhibitor upregulated SHMT2 (Figure 4D, E), and enhanced the proliferation and migration of these cells (Figure 4F, G). Western blot analyses showed that SHMT2 knockdown resulted in the downregulation of cyclinD1, Bcl-2, vimentin, and N-cadherin, and the upregulation of E-cadherin (Figure 4C), while upregulation of SHMT2 caused the opposite effects (Figure 4D). Consistent results were observed in the apoptosis assay (Figure 4H).

### Exogeneous SHMT2 reverses the effects of miR-383-5p on the proliferation and migration of LUAD cells

**Figure 5:**
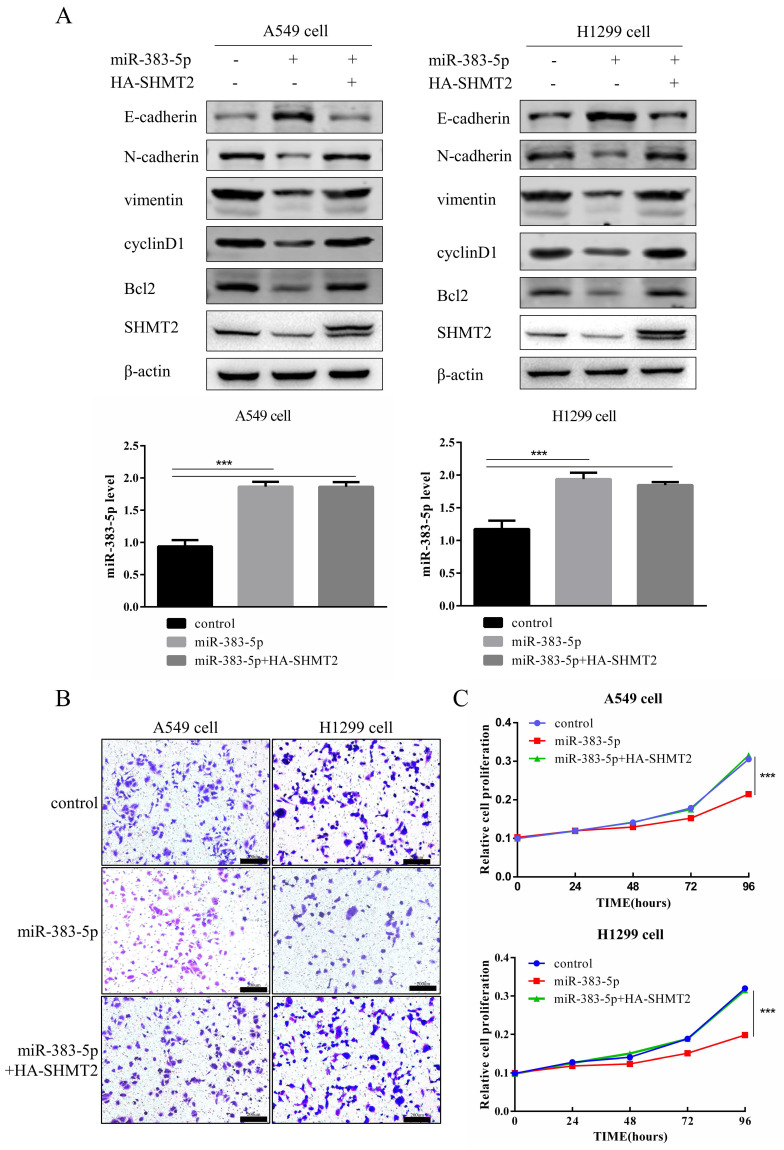

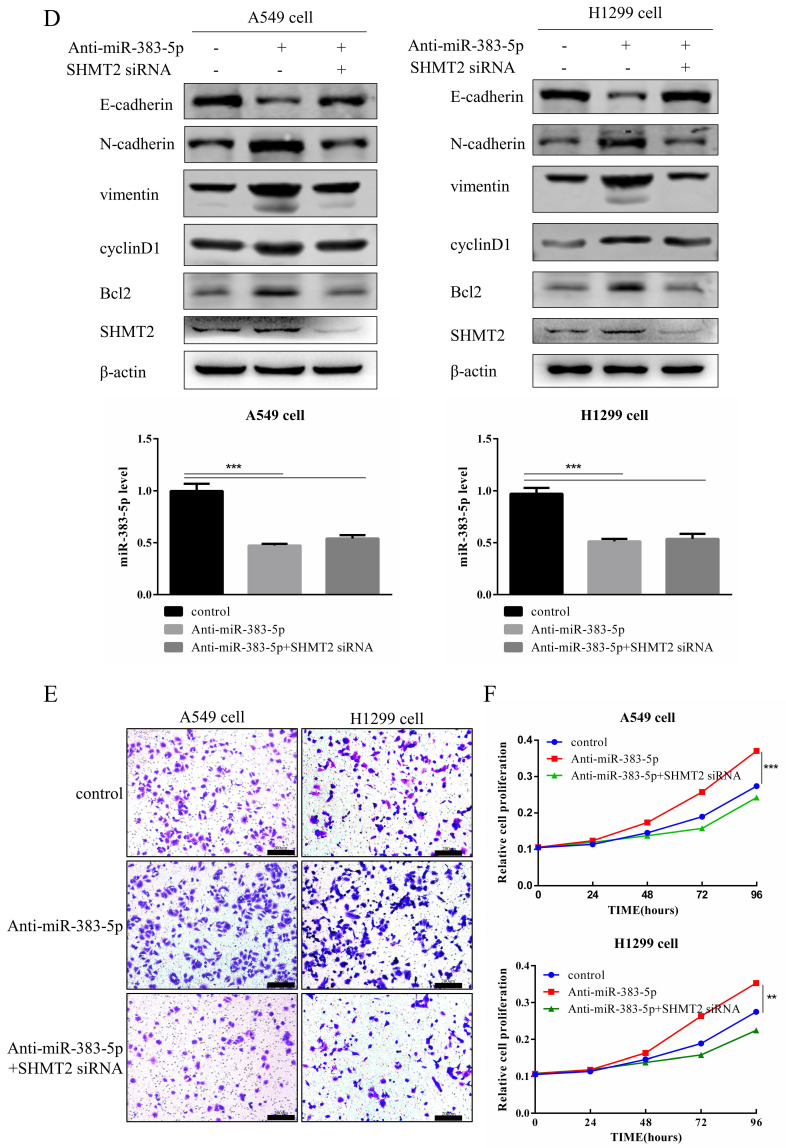
** MiR-383-5p suppresses the proliferation and migration of A549 and H1299 cells by inhibiting serine hydroxymethyltransferase 2 (SHMT2) expression. A.** A blank control, miR-383-5p mimic, or miR-383-5p mimic + HA-SHMT2 were transfected into A549 and H1299 cells. With β-actin as the control, the expression of SHMT2, Bcl-2, cyclinD1, and epithelial-mesenchymal transition (EMT)-associated proteins was detected via western blotting. miR-383-5p levels were detected using RT-qPCR. **B.** Migration of A549 and H1299 cells was detected using the Transwell assay. **C.** Proliferation of A549 and H1299 cells was detected using the Cell Counting Kit-8 (CCK-8) assay. **D.** A549 and H1299 cells were transfected with the blank control, miR-383-5p inhibitor (anti-miR-383-5p), or miR-383-5p inhibitor + SHMT2 siRNA. The expression of SHMT2, Bcl-2, cyclinD1, and EMT-associated proteins was detected using western blotting. β-actin was used as the control. **E.** Cell migration was detected using the Transwell assay. **F.** The proliferation of A549 and H1299 cells was detected using the CCK-8 assay. **P < 0.01, *** *P* < 0.001.

Next, we investigated whether miR-383-5p exerted proliferative and migratory effects on LUAD cells by regulating SHMT2. A549 and H1299 cells were transfected with the control, miR-383-5p mimic, or miR-383-5p mimic, and HA-tagged SHMT2. Western blotting results showed that the effect of miR-383-5p on E-cadherin, N-cadherin, vimentin, cyclinD1, and Bcl-2 could be restored by exogenous overexpression of SHMT2. Expression of the apoptotic protein, cyclinD1, and EMT-related proteins was restored after transfection with the SHMT2 plasmid (Figure 5A). Simultaneously, the CCK-8 proliferation and Transwell migration assays showed that the inhibitory effect of miR-383-5p on proliferation and migration was also restored (Figure 5B, C).

Finally, we transfected A549 and H1299 cells with the blank control, miR-383-5p inhibitor, or miR-383-5p inhibitor and SHMT2 siRNA. Western blotting results showed that transfection with the inhibitor increased the expression of SHMT2, N-cadherin, vimentin, cyclinD1, and Bcl-2. However, silencing SHMT2 with siRNA restored the effects of the miR-383-5p inhibitor on expression of the indicated proteins, and significantly weakened its impact on the proliferation and migration of A549 and H1299 cells (Figure 5D-F). Together, these results suggest that the effect of miR-383-5p on gene expression and cellular phenotype is related to SHMT2, and further confirmed that miR-383-5p targets SHMT2.

## Discussion

LUAD compromises the health of individuals worldwide. Despite the progress in various treatment methods in recent years, the survival rate remains poor, mainly because LUAD is difficult to detect in the early stages, often only being detected in the middle and late stages. Therefore, identifying markers and therapeutic targets for early diagnosis of LUAD is important for its treatment.

SHMT2 is highly expressed in various tumors and is associated with a poor prognosis. For example, SHMT2 is highly expressed in liver cancer, and its knockdown can inhibit the progression of this disease. According to prognostic analyses, patients with low SHMT2 levels have better outcomes and a higher overall survival rate 18. SHMT2 is also highly expressed in breast cancer and can be used as a prognostic indicator to assist in breast cancer treatment and diagnosis 19. Furthermore, high SHMT2 expression is associated with progression and poor prognosis of gastrointestinal tumors, and is a potential new target for the diagnosis and treatment of such tumors 15. Additionally, studies on bladder cancer have shown that SHMT2 overexpression promotes tumor cell growth and is associated with a poor prognosis 20. In the current study, an investigation using TCGA database showed that SHMT2 was highly expressed in LUAD tissue; therefore, we conducted an immunohistochemical analysis that confirmed this, and a survival analysis that showed SHMT2 was a factor for the poor prognosis of LUAD.

SHMT2 is a metabolic enzyme found primarily in mitochondria. It is recognized as a central hub coordinating cellular stress responses, mediating a variety of biological cellular processes including cell proliferation and apoptosis 21, 22. In this study, we analyzed the relationship between SHMT2, cyclinD1, and Bcl-2 in lung cancer. The results suggested that SHMT2 was positively correlated with cyclinD1 and Bcl-2 expression at both the protein and mRNA levels, and that SHMT2 knockdown led to the downregulation of cyclinD1 and Bcl-2. Liu et al. 17 reported that cytoplasmic SHMT2 interacts with and inhibits the ubiquitylation-mediated degradation of β-catenin and subsequently modulates the expression of its target genes. CyclinD1 is a downstream target of β-catenin. Therefore, we hypothesized that the effect of miR-383-5p on lung cancer cell proliferation is related to the upregulation of SHMT2 and β-catenin.

The EMT is an important biological process by which epithelial-derived malignant tumor cells acquire the ability to migrate and invade. Additionally, SHMT2 deficiency reportedly leads to prominent inhibition of invasion, metastasis, and the EMT in oral squamous cell carcinoma 23. In the present study, we found that SHMT2 knockdown also inhibited the EMT in lung cancer cell lines and had a notable influence on cell migration, indicating that SHMT2 is a tumor promoter.

Many studies have shown that miRNAs are closely associated with the occurrence and development of LUAD 24. Specifically, miR-383-5p acts as a tumor suppressor in various cancer types. For example, miR-383-5p inhibits the function of human malignant melanoma cells by targeting centromere protein F 25. Furthermore, miR-383-5p promotes ovarian granulosa cell apoptosis by targeting cold-inducible RNA-binding protein through the phosphoinositide 3-kinase/AKT signaling pathway 26. The upregulation of miR-383-5p inhibits the proliferation of ovarian cancer cells and enhances chemotherapy sensitivity by targeting TRIM27 27. Downregulating miR-383-5p stimulates the proliferation and migration of gastric cancer cells, and is associated with a poor prognosis 28.

In this study, the binding site of miR-383-5p with the SHMT2 3′-UTR was predicted by a TargetScan database analysis and confirmed by the luciferase reporter assay. We found that miR-383-5p overexpression substantially downregulated SHMT2, and reduced the proliferation and migration abilities of LUAD cells. In contrast, when miR-383-5p was inhibited, the expression level of SHMT2 increased, and the proliferation and migration abilities of LUAD cells increased. The effects of miR-383-5p on cell proliferation and migration could be restored by transfection of SHMT2 overexpression vectors without the 3′-UTR. Thus, we concluded that decreased expression of miR-383-5p in lung cancer cells is responsible for the upregulation of SHMT2 and lung cancer development. However, owing to the development of techniques for the early diagnosis of lung cancer, many patients with stage T1-3 N0-1 M0 lung cancer underwent surgery, and an insufficient number of specimens were collected to analyze the correlation between miR-383-5p and SHMT2.

## Conclusion

These results show that miR-383-5p inhibits the proliferation and migration of LUAD cells by downregulating SHMT2. Thus, miR-383-5p is a potential marker and a new target for the treatment of LUAD. Studies on the antitumor effects of SHMT2 inhibitors have entered the clinical experimental stage. With continued progress in miRNA delivery methods, miRNAs targeting *SHMT2*, such as miR-383-5p, may also have value as tumor-targeted therapeutic agents. Animal studies examining the effects of dysregulated SHMT2 and miR-383-5p expression on the growth and metastasis of lung cancer must be conducted in the future to verify this possibility.

## Abbreviations

CCK-8: Cell Counting Kit-8
SHMT2: Serine hydroxymethyl transferase 2
LUAD: Lung adenocarcinoma
miRNA: MicroRNA
SDS: Sodium dodecyl sulfate
PAGE: Polyacrylamide gel electrophoresis
RT-qPCR: Reverse transcription-quantitative polymerase chain reaction
PBS: Phosphate-buffered saline
TCGA: The Cancer Genome Atlas
EMT: Epithelial-mesenchymal transition
